# Cryptotanshinone Ameliorates Doxorubicin-Induced Cardiotoxicity by Targeting Akt-GSK-3β-mPTP Pathway In Vitro

**DOI:** 10.3390/molecules26051460

**Published:** 2021-03-08

**Authors:** Xiaoping Wang, Qianbin Sun, Qianqian Jiang, Yanyan Jiang, Yawen Zhang, Jing Cao, Linghui Lu, Chun Li, Peng Wei, Qiyan Wang, Yong Wang

**Affiliations:** 1 School of Traditional Chinese Medicine, Beijing University of Chinese Medicine, Beijing 100029, China; 18622961029@163.com (X.W.); 20200931144@bucm.edu.cn (Y.Z.); caojing20210701@163.com (J.C.); Lu-linghui@hotmail.com (L.L.); weipeng@bucm.edu.cn (P.W.); 2 Beijing Key Laboratory of TCM Syndrome and Formula, Beijing 100029, China; sunqianbin666@163.com (Q.S.); qianxixi0303@163.com (Q.J.); jiang__188199@163.com (Y.J.); lichun19850204@163.com (C.L.); 3 Key Laboratory of Beijing University of Chinese Medicine, Ministry of Education, Beijing 100029, China; 4 School of Life Sciences, Beijing University of Chinese Medicine, Beijing 100029, China; 5 School of Chinese Materia Medica, Beijing University of Chinese Medicine, Beijing 100029, China

**Keywords:** cardiotoxicity, doxorubicin, cryptotanshinone, oxidative stress, apoptosis, mPTP, GSK-3β

## Abstract

Cardiotoxicity is one of the main side effects of doxorubicin (Dox) treatment. Dox could induce oxidative stress, leading to an opening of the mitochondrial permeability transition pore (mPTP) and apoptosis in cardiomyocytes. Previous studies have shown that Cryptotanshinone (Cts) has potential cardioprotective effects, but its role in Dox-induced cardiotoxicity (DIC) remains unknown. A Dox-stimulated H9C2 cell model was established. The effects of Cts on cell viability, reactive oxygen species (ROS), superoxide ion accumulation, apoptosis and mitochondrial membrane potential (MMP) were evaluated. Expressions of proteins in Akt-GSK-3β pathway were detected by Western blot. An Akt inhibitor was applied to investigate the effects of Cts on the Akt-GSK-3β pathway. The effects of Cts on the binding of p-GSK-3β to ANT and the formation of the ANT-CypD complex were explored by immunoprecipitation assay. The results showed that Cts could increase cell viability, reduce ROS levels, inhibit apoptosis and protect mitochondrial membrane integrity. Cts increased phosphorylated levels of Akt and GSK-3β. After cells were co-treated with an Akt inhibitor, the effects of Cts were abolished. An immunoprecipitation assay showed that Cts significantly increased GSK-3β-ANT interaction and attenuated Dox-induced formation of the ANT-CypD complex, thereby inhibiting opening of the mPTP. In conclusion, Cts could ameliorate oxidative stress and apoptosis via the Akt-GSK-3β-mPTP pathway.

## 1. Introduction

Doxorubicin (Dox) is an effective first-line chemotherapeutic agent that is widely used in the treatment of various kinds of cancers. However, accumulation of Dox can cause side effects, cardiotoxicity being the most serious one [1]. Currently, there are few effective drugs that can be used to prevent Dox-induced cardiotoxicity (DIC) and the underlying mechanism of DIC remains poorly understood.

Mitochondrial dysfunction is one of the major pathogenic changes caused by DIC. Administration of Dox could induce oxidative stress, and subsequently lead to the opening of the mitochondrial permeability transition pore (mPTP) in cardiomyocytes [2,3,4]. The caspase-dependent intrinsic apoptotic pathway is then activated, as apoptogenic cytochrome c is released into the cytoplasm through the mPTP opening [5]. Therefore, modulating mPTP opening-induced apoptosis is a potential way to ameliorate cardiotoxicity caused by Dox.

The mPTP, comprised of Bax/Bak, adenine nucleotide translocator (ANT), cyclophilin D (CypD) and a phosphate carrier, is a polyprotein complex embedded in mitochondrial membranes [6]. Reactive oxygen species (ROS) within mitochondria may trigger mPTP opening which is associated with further stimulation of ROS formation [7]. Opening of the mPTP will eventually trigger cell death and is a key event in the process of cardiotoxicity. Opening of the mPTP is regulated by CypD and glycogen synthase kinase 3β (GSK-3β), which is a protein kinase that has been reported to be involved in cardiovascular diseases [8]. It has been shown that GSK-3β stimulates the mPTP opening, thereby inducing mitochondrial dysfunctions and apoptosis [9,10]. Meanwhile, inhibition of GSK-3β protects against cardiotoxicity by desensitizing the mPTP [11]. Previous studies have shown that increasing phosphorylation of GSK-3β at serine 9 (ser 9) could promote its interaction with ANT, thereby inhibiting the opening of the mPTP and preventing apoptosis [12]. Therefore, it is feasible to attenuate apoptosis and protect the integrity of mitochondria by increasing phosphorylation of GSK-3β.

Cryptotanshinone (Cts) is one of the major bioactive constituents isolated from *Salvia miltiorrhiza*, which is a widely used herbal medicine for the treatment of cardiovascular diseases [13]. It has been shown that Cts has cardioprotective properties against cardiac fibrosis, inflammatory response, mitochondrial dysfunction and apoptosis [14,15,16,17,18,19]. Some studies reported that Cts has potential cardioprotective effects against DIC. However, little is known about the underlying protective mechanisms of Cts. In this study, an in vitro DOX-stimulated H9C2 cell model was established. The protective effects of Cts against oxidative stress and apoptosis were explored. In particular, the effects of Cts on GSK-3β and mPTP opening were comprehensively investigated. This study will provide potential targets of DIC and offer alternative therapeutic strategies for the clinical management of DIC.

## 2. Results

### 2.1. Effects of Cts on Mitochondrial Oxidative Stress

**Figure 1:**
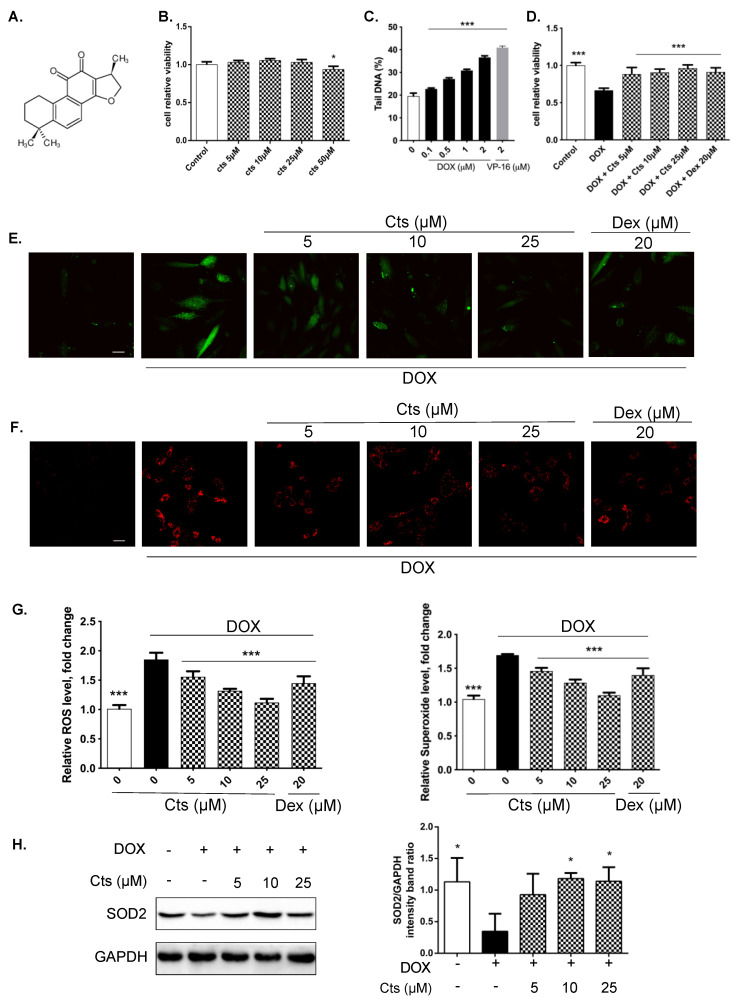
Effects of Cts on mitochondrial oxidative stress. (**A**) Chemical structure of Cts. (**B**) CCK-8 assay showed that Cts did not reduce cellular viability at concentrations of 5–25 μM. Cellular viability was reduced by 6.3% at a concentration of 50 μM. Results were presented as means ± SD (*n* = 6 per group). * *p* < 0.05 vs. control group. (**C**) The extent of double-stranded DNA breaks induced by Dox in H9C2 cells was determined by a neutral comet assay following 24 h of treatment. Results were presented as means ± SD (*n* = 6 per group). *** *p* < 0.001 vs. Dox-treatment group. (**D**) Dox treatment reduced cellular viability by 33.3% and Cts and Dex treatment both significantly up-regulated cellular viability. Results were presented as means ± SD (*n* = 6 per group). *** *p* < 0.001 vs. Dox-treatment group. (**E**) Accumulation of H_2_O_2_ in H9C2 cells was detected by probes DCFH2-DA and representative images showed that Dox treatment induced production of H2O2. Cts and Dex treatment both reduced cellular levels of H_2_O_2_. Scale bar, 50 μm. (**F**) Accumulation of superoxide ions in H9C2 cells was detected by probes DHE and representative images showed that Dox treatment induced production of superoxide ions and Cts and Dex treatment both reduced cellular levels of superoxide ions. Scale bar, 50 μm. (**G**) Quantification of relative H_2_O_2_ levels and superoxide ion levels in H9C2 cells. Fluorescence intensity was determined by image J software. Results were presented as means ± SD (*n* = 3 repeated experiments, 15 cells per group). *** *p* < 0.001 vs. Dox-treatment group. (**H**) Western blot showed that the SOD2 level was significantly reduced by 39.2% in the Dox-treated group compared with the control group. Cts treatment at concentrations of 10 μM and 25 μM significanlty up-regulated the expression of SOD2. Results were presented as means ± SD from independent experiments performed in triplicate. * *p* < 0.05 vs. Dox-treatment group.

The structure of Cts is shown in Figure 1A. The effect of Cts on the viability of H9C2 cells was measured by a CCK-8 assay. Under normal conditions, Cts at a concentration of 25 μM did not reduce cell viability over a period of 48 h, indicating that Cts doesn’t cause cellular toxicity (Figure 1B). In our previous study, a Dox-induced H9C2 cell injury model was established and a 1 μM Dox dose was applied [20]. Furthermore, the induction of double-strand DNA breaks arising from the treatment of H9C2 cells with Dox was assessed by a neutral comet assay. Results showed that Dox produced significant DNA breaks in a dose-dependent manner, as measured by percent tail DNA after treatment. Cts protected against Dox-induced cellular injury in a dose-dependent manner (Figure 1C).

The effect of Cts on the intracellular redox status was determined by measuring levels of H_2_O_2_ and superoxide ions. Dox dramatically increased the production of intracellular H_2_O_2_ and superoxide ions by 85% and 69%, respectively (Figure 1D–F). After cells were co-treated with Cts, Dox-induced ROS were significantly reduced, indicating that Cts has antioxidant effects (Figure 1D–F). Furthermore, Cts has better antioxidant effects compared with Dexrazoxane (Dex).

SOD2 is the primary antioxidant enzyme neutralizing •O^2−^ in mitochondria and serves as a first line of defense to protect the mitochondria in physiological and pathophysiological conditions [21]. Western blot results showed that there was an approximately 80% decrease in the level of SOD2 in cells treated with Dox compared to the control group, while Cts treatment increased SOD2 expression, further demonstrating that Cts protects against oxidative stress (Figure 1G).

### 2.2. Effects of Cts on Mitochondrial Apoptosis

**Figure 2:**
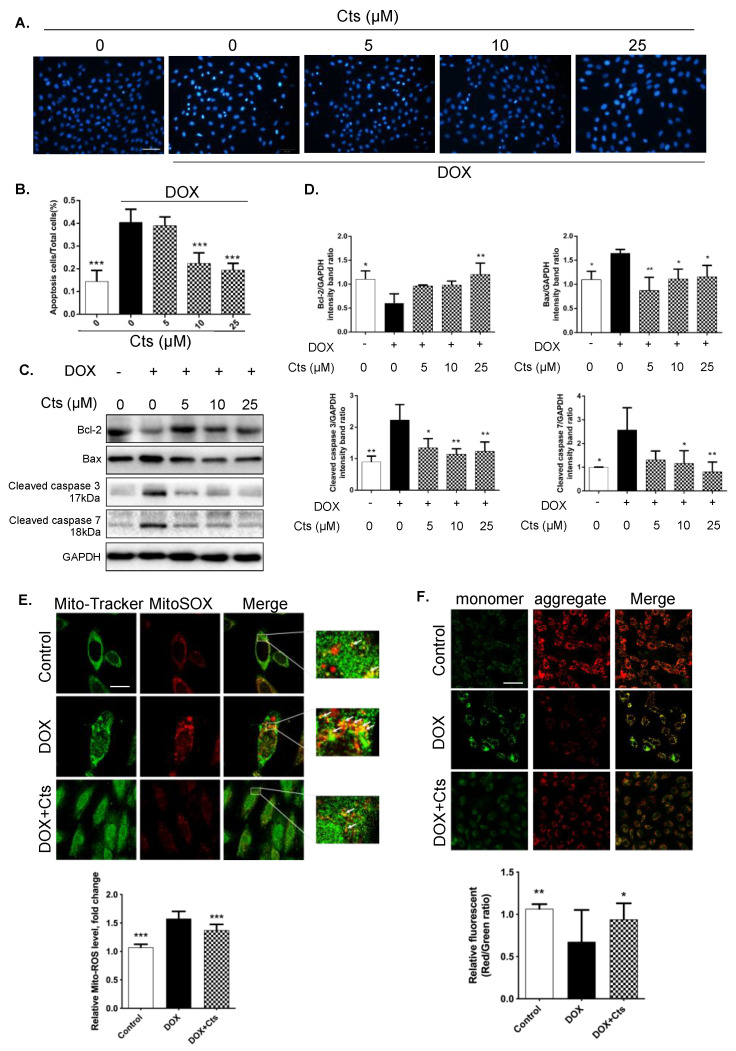
Effects of Cts on mitochondrial apoptosis. (**A**) The Hoechst 33342 staining showed that apoptosis increased in the Dox-treated group, whereas Cts protected cells against Dox-induced apoptosis. Scale bar, 20 μm. (**B**) Fluorescence intensity of Hoechst staining was determined by image J software. Results were presented as means ± SD (*n* = 3 repeated experiments, 15 cells per group). *** *p* < 0.001 vs. Dox-treatment group. Results showed that Dox increased the apoptotic rate by 40.4%. Cts at 10 μM and 25 μM significantly reduced apoptotic rates by 22.4% and 19.4%, respectively. (**C**,**D**) Cts regulated expressions of proteins involved in apoptosis, including Bcl-2, Bax, Cleaved caspase 3 and Cleaved caspase 7. (**E**) Representative images of Mito-tracker, MitoSOX and merged images of both. Quantification of yellow dots in merged images. Scale bar, 50 μm. (**F**) Representative images of JC-1 aggregates, JC-1 monomers and merged images of both. Ratio of JC-1 aggregates to JC-1 monomers. Scale bar, 25 μm. Results were presented as means ± SD from independent experiments performed in triplicate. * *p* < 0.05, ** *p* < 0.01 vs. Dox-treatment group.

Hoechst 33342 staining was carried out to determine the effect of Cts on cellular apoptosis. More condensed, fragmented and highlighted fluorescence was observed in the Dox-treated group, indicating higher level of apoptosis (Figure 2A). Cts treatment at concentrations of 10 μM and 25 μM significantly reduced apoptotic rates by 22.4% and 19.4%, respectively, demonstrating an anti-apoptotic property (Figure 2A,B).

Western blot results showed that the expression of Bcl-2 was down-regulated while the expressions of Bax, Cleaved caspase 3 (Casp3) (cleaved at Asp175) and Cleaved caspase 7 (Casp7) (cleaved at Asp198) were upregulated in the Dox-treated group compared to the control group (Figure 2C,D). Interestingly, treatment with Cts regulated expressions of apoptotis-related proteins back towards normal levels (Figure 2C,D). In additon, we carried out fluorescent staining and analysis of Mito-morphology (MitoTracker) and intra-Mito ROS (MitoSox). Results showed that compared to control group, Dox induced an increased level of intro-Mito ROS, while Cts treatment could dramatically counteract the situation (Figure 2E). Furthermore, we measured mitochondrial membrane potential by JC-1. Results showed that the JC-1 aggregate was converted to the JC-1 monomer after Dox treatment. Compared to the control group, Dox treatment increased the JC-1 monomer amount as determined by the corresponding increase in green fluorescence intensity. While Cts treatment counteracted the situation, indicating that Cts prevented a decrease in the ΔΨm (Figure 2F).

### 2.3. Effects of Cts on Mitochondrial Membrane Integrity and Phosphorylation of Akt and GSK-3β

**Figure 3:**
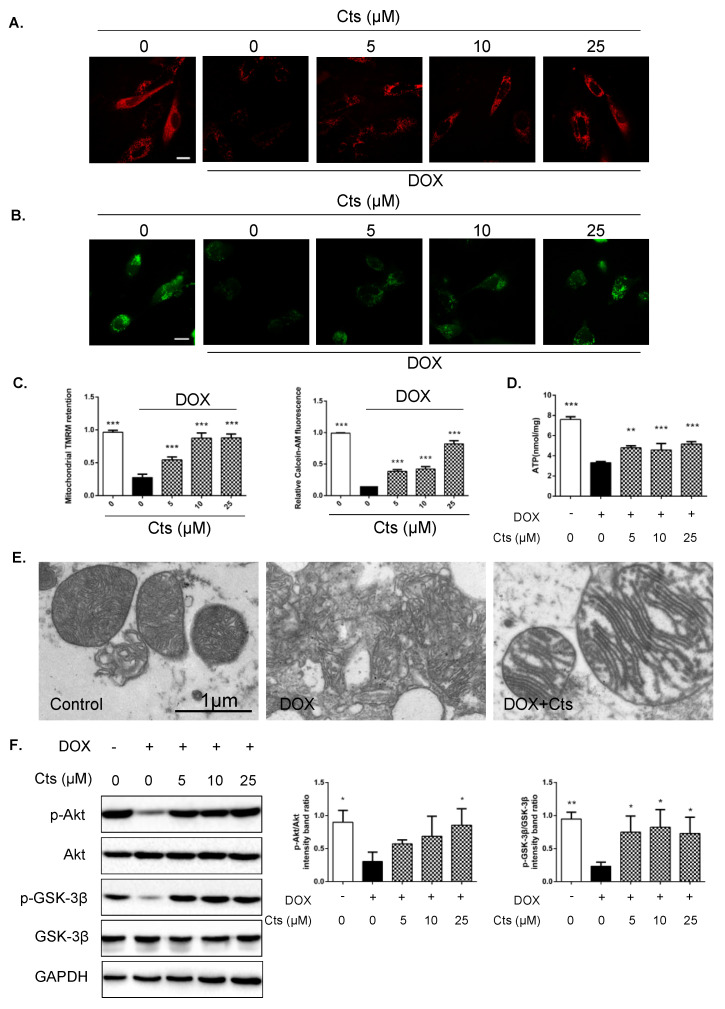
Effects of Cts on mitochondrial membrane integrity and phosphorylation of Akt and GSK-3β. Fluorescence images of tetramethylrhodamine methyl ester perchlorate (TMRM) sequestration (**A**) and calcein sequestration (**B**) in mitochondria. Scale bar, 50 μm. (**C**) Quantification of TMRM sequestration and calcein sequestration in mitochondria. Fluorescence intensity was determined by image J software. Results were presented as means ± SD (*n* = 3 repeated experiments, 15 cells per group). *** *p* < 0.001 vs. Dox-treatment group. (**D**) Dox decreased ATP production. Cts at different concentrations significantly increased ATP levels. Results were presented as means ± SD (*n* = 6 per group). ** *p* < 0.01, *** *p* < 0.001 vs. Dox-treatment group. (**E**) Electron microscopy was used to compare mitochondrial morphology in cardiomyocytes from different groups. (**F**) Western blot showed that the levels of p-AKT and p-GSK-3β were significantly increased in the Cts-treatment group compared with the Dox-treatment group. Results were presented as means ± SD from independent experiments performed in triplicate. * *p* < 0.05 vs. Dox-treatment group.

To determine the effect of Cts on mitochondrial membrane integrity, cationic dye tetramethylrhodamine methyl ester (TMRM) was applied to assess mitochondrial membrane potential (MMP) [22]. Active and intact mitochondria can sequester TMRM, which emits red fluorescence. As shown in Figure 3A, after exposure to Dox for 24 h, the capability of mitochondria to sequester TMRM was significantly diminished relative to the untreated control cells, indicating that mitochondrial membrane integrity was damaged by Dox treatment. Cts treatment preserved MMP in a dose-dependent manner (Figure 3A,C). Calcein-AM staining showed similar results (Figure 3B,C). Maintenance of mitochondrial membrane potential is essential for the generation of adenosine triphosphate (ATP). Dox treatment significantly reduced production of ATP in H9C2 cells, whereas Cts treatment restored the level of ATP (Figure 3D). Furthermore, compared to the control group, there was a perturbation of mitochondrial membrane permeability in the Dox-treated group, while Cts treatment preserved the integrity of mitochondrial membrane (Figure 3E).

GSK-3β plays an important role in regulating MMP. Akt inactivates GSK-3β by phosphorylating it at serine 9 [23]. The effects of Cts on GSK-3β and Akt were explored. As shown in Figure 3E, the levels of p-Akt and p-GSK3β (Ser9) were suppressed under Dox-stimulation. Interestingly, Cts treatment increased phosphorylated levels of Akt and GSK-3β, suggesting that the protective effect of Cts might be exerted through acting on the GSK-3β pathway (Figure 3F).

### 2.4. Cts Protected against Dox-Induced Mitochondrial Injury by Activating Akt

**Figure 4:**
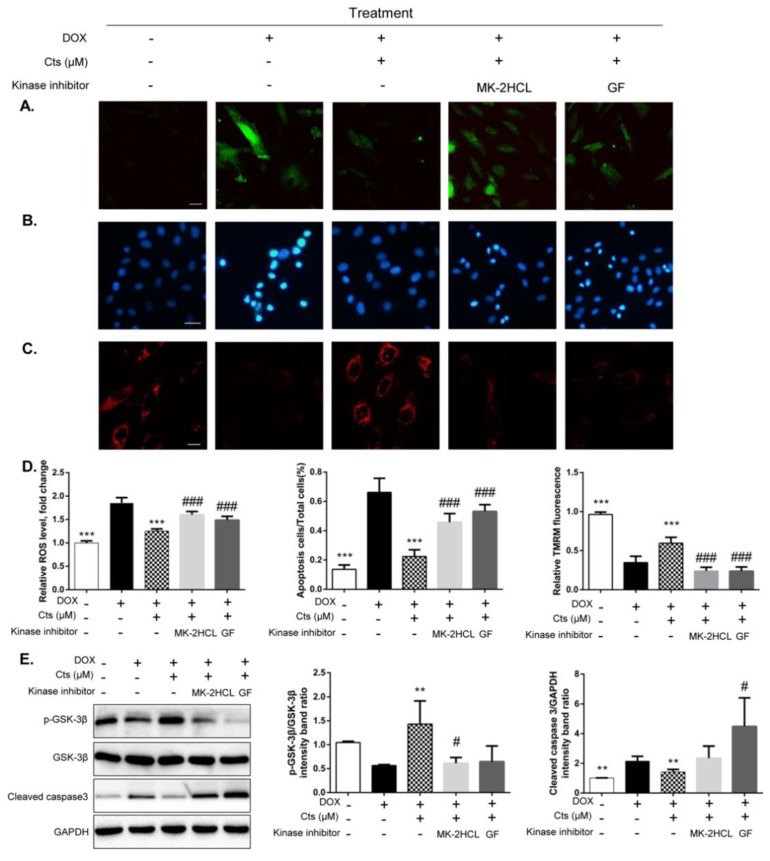
Cts protected against Dox-induced mitochondrial injury via the AKT-GSK-3β signaling pathway. (**A**) Accumulation of H_2_O_2_ in H9C2 cells was detected by probes DCFH2-DA. MK-2HCL abolished the inhibitory effect of Cts on H_2_O_2_. Scale bar, 50 μm. (**B**) Hoechst 33342 staining showed that MK-2HCL abolished the effect of Cts on apoptosis. Scale bar, 20 μm. (**C**) TMRM sequestration assays showed that MK-2HCL abolished the protective effect of Cts on MMP. Scale bar, 50 μm. (**D**) Quantification of fluorescence including DCFH-DA, apoptotic cells and TMRM. Fluorescence intensity was determined by Image J software. Results were presented as means ± SD (*n* = 3 repeated experiments, 15 cells per group). *** *p* < 0.001 vs. Dox-treatment group, ### *p* < 0.001 vs. Cts-treatment group. (**E**) Western blot showed that the effects of Cts on p-AKT and p-GSK-3β were significantly abolished by Akt inhibitor. Results were presented as means ± SD from independent experiments performed in triplicate. ** *p* < 0.01, *** *p* < 0.001 vs. Dox-treatment group, # *p* < 0.05, ### *p* < 0.001 vs. Cts-treatment group.

To confirm that Cts protected against Dox-induced injury through phosphorylation of GSK-3β at the site of Ser 9, Akt inhibitor MK-2HCL was applied to treat H9C2 cells. As shown in Figure 4A–D, MK-2HCL antagonized the effects of Cts on ROS production, apoptosis, as well as mitochondrial membrane integrity. The effect of Cts on cleaved caspase 3 was also abolished (Figure 4E). After cells were co-treated with Akt inhibitor, the effect of Cts on phosphorylation of GSK-3β at Ser 9 was also abolished (Figure 4E).

### 2.5. Cts Stimulated the Binding of p-GSK-3β to ANT and Inhibited the Formation of ANT-CypD Complex in mPTP

**Figure 5:**
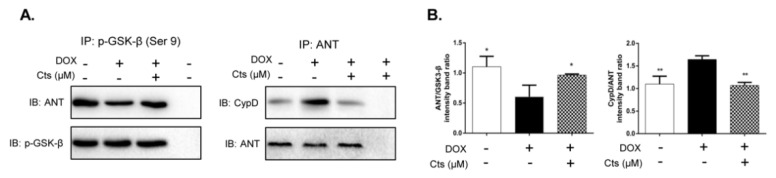
Effects of Cts on the binding of p-GSK-3β (Ser9) to ANT and the formation of the ANT-CypD complex. (**A**) Immunoprecipitation assay showed that Dox stimulation decreased binding of p-GSK-3β to ANT, whereas Cts treatment increased p-GSK-3β-ANT interaction. (**B**) Immunoprecipitation assay showed that formation of the CypD-ANT complex was activated by Dox-stimulation. Cts treatment inhibited interaction of CypD and ANT. Results were presented as means ± SD from independent experiments performed in triplicate. * *p* < 0.05, ** *p* < 0.01 vs. Dox-treatment group.

To further explore the effect of Cts on the mPTP opening, binding of phospho-GSK-3β (Ser9) to ANT was explored. Meanwhile, Cyp-D-ANT interaction was also investigated by immunoprecipitation. Compared to the control group, Dox-stimulation decreased the binding of phospho-GSK-3β (Ser9) to ANT, whereas Cts treatment significantly increased the phospho-GSK-3β (Ser9)-ANT interaction (Figure 5A). Meanwhile, Dox-stimulation increased the formation of the ANT-Cyp-D complex, whereas Cts attenuated Dox-induced formation of the ANT-Cyp-D complex (Figure 5B).

## 3. Discussion

**Figure 6:**
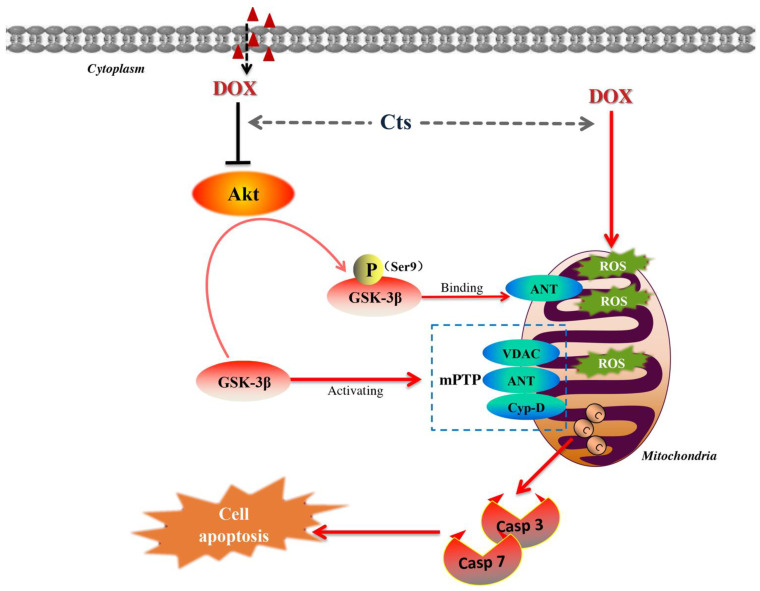
The effects of Cryptotanshinone (Cts) on the Akt-GSK-3β-mPTP signaling pathway. The level of reactive oxygen species (ROS) was up-regulated by Doxorubicin (Dox)-stimulation. Cts could reduce ROS level, down-regulate caspase 3 (Casp3) and caspase 7 (Casp7) activity, and inhibit cell apoptosis. The mitochondrial permeability transition pore (mPTP) is made of three key components, including ANT, CypD and VDAC. Cts could rescue the opening of mPTP by inhibiting CypD-ANT interaction and increasing p-GSK-3β-ANT interaction. In conclusion, Cts could ameliorate oxidative stress and apoptosis via the Akt-GSK-3β-mPTP pathway.

Cardiotoxicity is one of most serious side effects induced by Dox. Presently, Dexrazoxane is the only drug that has been approved by the FDA for the prevention and treatment of DIC [24]. Furthermore, it has been reported that metformin would be a preferable drug to help cardiomyocytes survive DIC [25]. Investigation of DIC pathogenesis and exploration of effective drugs have great clinical significance. The main findings of this study indicates that Cts could ameliorate Dox-induced oxidative damage and apoptosis by inhibiting mPTP opening through the Akt-GSK-3β pathway (Figure 6).

A cardiomyocyte injury model was established by Dox stimulation. Cellular viability was significantly reduced by Dox treatment, the level of reactive oxygen species was up-regulated and the apoptotic rate was increased by Dox stimulation. Furthermore, mitochondrial membrane integrity was damaged and ATP production was decreased after cells were treated with Dox. These data demonstrated that mitochondrial dysfunction induced by oxidant stress is an important pathogenesis of DIC. Previous studies have shown that Dox could increase ROS both in vivo and in vitro [26,27]. ROS triggers the opening of the mPTP on the mitochondrial membrane, leading to further mitochondrial damage and eventual cell death [3]. Therefore, inhibiting mPTP opening and protecting the integrity of mitochondria could be important strategies for the prevention and treatment of DIC. Herbal medicine has been widely used in the management of cardiovascular diseases and active components of some effective herbs serve as candidates for therapeutics of DIC. Cryptotanshinone is one of major components of salvia miltiorrhiza and previous studies have shown that tanshinones have cardio-protective efficacies [19,28]. In this study, we explored the pharmacological mechanism of Cts against DIC. Caspase activation is a major event in apoptosis [29]. Inhibition of caspases may be therapeutically beneficial to reduce stress-induced apoptotic cell death [30]. Our results showed that Cts could improve cellular viability, reduce ROS levels, down-regulate caspase activity, inhibit apoptosis and rescue the loss of mitochondrial membrane potential.

The effects of Cts on mPTP-induced mitochondrial dysfunction were further investigated. The mPTP is made of three key components, including ANT, CypD and VDAC. Under normal conditions, the mPTP is closed. The opening of mPTP is triggered under stressful conditions, such as oxidative stress, calcium overload and adenine nucleotide deficiency [31]. Binding CypD to ANT will open the mPTP and release cytochrome c, leading to apoptosis. Molecules less than 1500 Da will also pass through the mPTP into the mitochondria, leading to a swelling of the mitochondria and disruption of mitochondrial membrane potential once the mPTP is opened. Therefore, suppressing CypD-ANT interaction will attenuate the opening of the mPTP. Phosphorylated-GSK-3β has been shown to interact with ANT, leading to the inhibition of CypD-ANT interaction [32]. Studies have demonstrated that increasing phosphorylation of GSK-3β will inhibit mPTP opening and protect mitochondrial function [12,32]. In this study, the results showed that DOX treatment could reduce the level of p-GSK-3β, leading to opening of the mPTP, loss of mitochondrial membrane potential and increased apoptotic rates. After cells were co-treated with Cts, the level of p-GSK-3β increased. The level of Akt, which phosphorylates and inactivates GSK-3β, was also increased by Cts treatment. These data suggests that Cts protects against DIC through inhibiting the GSK-3β-dependent mPTP opening pathway.

To further confirm that Cts inhibits mPTP opening by increasing phosphorylation of GSK-3β, an Akt inhibitor, which suppresses the phosphorylation of GSK-3β, was applied to treat cells. After cells were treated the Akt inhibitor, the protective effects of Cts were abrogated. Moreover, an immunoprecipitation assay showed that phospho-GSK-3β-ANT interaction was enhanced by Cts. Binding of ANT with phosphorylated GSK-3β suppresses ANT-CypD interaction and inhibits mPTP opening. Indeed, the formation of ANT-CypD complex was reduced by Cts. Taken together, Cts protected mitochondrial function by increasing phosphorylation of GSK-3β and inhibiting mPTP opening.

## 4. Materials and Methods

### 4.1. Cell Culture and Cell Viability

Rat cardiomyocyte H9C2 cell line (Cell Resource Center, IBMS, CAMS/PUMC, Beijing, China) was cultured in Dulbecco’s modified Eagle’s medium (DMEM) containing 10% heat-inactivated fetal bovine serum (FBS) in a 37 °C humidified atmosphere supplemented with 95% O2 and 5% CO2. The Dox-induced H9C2 cell injury model has been reported in our previous study [20]. Briefly, H9C2 cells (6000 cells/well) were cultured in a 96-well plate. After incubation with Cts (5 μM, 10 μM and 25 μM) for 24 h, cells were cotreated with 1 µM DOX for another 24 h. Subsequently, a Cell Counting Kit-8 (CCK-8, Dojindo, Kumamoto, Japan) was used to assess cell viability. The original medium was discarded and CCK-8 in DMEM solution at a ratio of 10% was added to the plate for two hours at 37 °C. Finally, using a microplate reader (Perkin-Elmer, Waltham, MA, USA), the absorbance was detected at 450 nm.

### 4.2. Detection of Reactive Oxygen Species (ROS) and Superoxide Production

H9C2 cells were seeded into glass-bottom cell culture dishes (20 mm) at a density of 4 × 10^4^ cells/mL. After 24 h incubation, the cells were exposed to Cts at different concentrations for 24 h and subsequently cotreated with Dox for 24 h. For the measurement of intracellular ROS, the cells were stained with 10 μM 2′,7′-Dichlorofluorescin-diacetate (DCFH2-DA, Sigma-Aldrich LLC, China) at 37 °C for 30 min. The production of intracellular superoxide was detected with dihydroethidium (DHE, Sigma-Aldrich LLC, China) at 37 °C for 30 min. Once entered into the cells, the nonpolar probe DCFH2-DA was transformed into the polar intermediate DCFH by cellular esterase and then oxidized to a highly fluorescent 2′,7′-dichlorofluorescein (DCF) by H_2_O_2_ and other types of ROS. In addition, a red fluorescent product, 2-hydroxyethidium (EOH), was generated when superoxide anion reacted with DHE. The cells were imaged on a laser scanning fluorescence microscope (Olympus, BX50-FLA, Tokyo, Japan). The results were presented as a percentage of the intracellular ROS or superoxide production by using Image J 1.8.0 software https://imagej.nih.gov/ij/(accessed on 5 January 2020).

### 4.3. Hoechst Staining

H9C2 cells were seeded into a 12–well plate at a density of 3 × 10^4^ cells/mL. After being treated by different culture solutions, cells were incubated with phosphate buffer saline (PBS) containing 10 μg/mL Hoechst 33342 (Beijing BioDee Biotechnology Co., Ltd., Beijing, China) for 20 min in the dark at 37 °C. Then H9C2 cells were washed 3 times with PBS. Finally, cellular apoptosis was observed under a fluorescence microscope (Olympus, BX50-FLA, Tokyo, Japan).

### 4.4. Measurement of Mitochondrial Membrane Integrity

Mitochondrial membrane integrity was measured by staining cells with tetramethylrhodamine methyl ester (TMRM, Sigma-Aldrich, Shanghai, China) and calcein-AM (Sigma-Aldrich, Shanghia, China) as reported [33]. TMRM is a cell-permeant, cationic and red-orange fluorescent dye that is readily sequestered by active mitochondria. Calcein-AM is a non-fluorescent molecule that accumulates in cytoplasm and mitochondria. Cleavage of calcein-AM by intracellular esterase releases fluorescent calcein dye that does not cross cytomembrane or mitochondrial membrane. In normal cells, addition of cobalt chloride hexahydrate quenches the fluorescence of cytosolic but not mitochondrial calcein. In cells with the mPTP opening, cobalt chloride hexahydrate also enters mitochondria to quench the mitochondrial calcein fluorescence. After being treated with different culture solutions, H9C2 cells were incubated with 250 nM TMRM in DMEM for 30 min. For calcein-AM staining, H9C2 cells were treated with 1 μM calcein-AM in the presence of 8 mM Cobalt chloride hexahydrate (Shanghai YuanYe Bio-Technology Co., Ltd., Shanghai, China) for 30 min. After being washed three times with PBS, the cell monolayer was imaged under a fluorescence microscope.

### 4.5. Western Blot Analysis

Cells were lysed in pre-cold RIPA buffer (Beijing Pulilai Gene Technology Co., Ltd., Beijing, China) containing 1% protease inhibitor (Beijing Pulilai Gene Technology Co., Ltd., Beijing, China). The sample was loaded on the 10% SDS-PAGE gel and transferred onto the polyvinylidene fluoride (PVDF) membrane. Then the membrane was incubated with the primary antibody at 4 °C overnight, and subsequently incubated with a secondary antibody at room temperature for 1 h. Finally, after reacting with electrochemiluminescence (ECL) (Beijing PuLilai Gene Technology Co., Ltd., Beijing, China) for one minute in the dark, all bands on the membrane were detected and analyzed using Image Lab software (http://www.imagelab.at/ (accessed on 2 April 2020)). The following antibodies were used: anti-phospo-Akt (CST4060S, Cell Signaling Technology, Beverly, MA, USA), anti-Akt (CST4691S, Cell Signaling Technology, Beverly, MA, USA), anti-phospo-GSK3β (CST5558S, Cell Signaling Technology, Beverly, MA, USA), anti-GSK3β (CST12456T, Cell Signaling Technology, Beverly, MA, USA), anti-Bcl-2 (ab7973, Abcam, UK), anti-Bax (ab32503, Abcam, UK), anti-Cleaved caspase3 (CST9664, Cell Signaling Technology, Beverly, MA, USA), anti-Cleaved caspase7 (CST8438, Cell Signaling Technology, Beverly, MA, USA), anti-GAPDH (CST5174S, Cell Signaling Technology, Beverly, MA, USA) and anti-Rabbit IgG H&L (ab16284, Abcam, UK).

### 4.6. Immunoprecipitation (IP)

IP was performed as previously reported [34]. Briefly, 100 μg of total cell lysates from different treatment groups were incubated with 1 μg phospho GSK-3β (Cell Signaling Technology, Beverly, MA, USA) or ANT (Thermo Fisher Scientific Inc., Waltham, MA, USA) for 1 h at 4 °C. Then 20 μL protein A Agarose Beads (CST9863T, Cell Signaling Technology, Beverly, MA, USA) were added and co-incubated overnight at 4 °C. Subsequently, by centrifuging at 3000× *g* rpm for 5 min at 4 °C, the precipitations of samples were recovered and washed three times with PBS. Finally, the pellets were dissolved in gel loading buffer and detected by sodium dodecyl sulfate polyacrylamide gel electrophoresis (SDS–PAGE). Thirty microliters of each sample were analyzed by Western blot. The pull-down materials were analyzed by immunoblotting (IB) with antibodies against ANT and Cyp-D (Santa Cruz, CA, USA).

### 4.7. Comet Assay

The neutral comet assay was performed as previously described [35]. Percentage of DNA in the tail was calculated as previously described [36].

### 4.8. JC-1 Assay

H9C2 cells were seeded into a 6–well plate at a density of 3 × 10^4^ cells/mL. After different treatments, cells were harvested and 2 mL DMEM was added to prepare for cell suspension. Then, JC-1 detection solution was added for chemical reaction in the dark for 10 min. A spectrofluorometer was implemented for detecting the fluorescence intensity (590 nm/527 nm) according to the instructions.

### 4.9. Statistical Analysis

All results were presented as the means ± SD from three independent experiments. For multiple comparisons between groups, statistical analysis was carried out by Dunnett’s test and one-way analysis of variance (ANOVA) using GraphPad Prism 5.0 software (https://www.graphpad.com/scientific-software/prism/ (accessed on 12 September 2020)). *p* < 0.05 was considered as statistically significant.

### 4.10. Materials and Reagents

Cts and Cobalt chloride hexahydrate were obtained from Shanghai YuanYe Bio-Technology Co., Ltd. (Shanghai, China). DCFH2-DA, DHE, TMRM and calcein-AM were obtained from Sigma-Aldrich LLC (Shanghai, China). VP-16 was purchased from MCE (Shanghai, China). Antibodies for phospho-GSK-3β, GSK-3β, phospho-Akt, Akt, Cleaved caspase 3, Cleaved caspase 7 and GAPDH were obtained from Cell Signaling Technology (Boston, MA, USA). Antibodies for Bax, Bcl-2 and Rabbit IgG H&L were purchased from Abcam (Cambridge, UK). Antibody for ANT was purchased from Thermo Fisher Scientific Inc. (Waltham, MA, USA). Antibody for CypD was purchased from Santa Cruz (CA, USA). CCK-8 was purchased from Dojindo (Kumamoto, Japan).

## 5. Conclusions

In conclusion, cryptotanshinone is an effective agent for protection against Dox-induced cardiotoxicity. Cryptotanshinone reduces oxidative stress, inhibits apoptosis and preserves mitochondrial integrity. The protective effects may be exerted through up-regulating phosphorylation of GSK-3β, which subsequently inhibits mPTP opening. This study demonstrates that components of herbal medicine are valuable sources for the development of novel drugs and the study provides potential targets for the management of DIC.

## Data Availability

The majority of the data can be found in the manuscript, and further data used to support the findings of this study are available from the corresponding author upon reasonable request.

## Sample Availability

Sample of the compound (cryptotanshinone) is available from the authors.
